# Effects of the K65R and K65R/M184V reverse transcriptase mutations in subtype C HIV on enzyme function and drug resistance

**DOI:** 10.1186/1742-4690-6-14

**Published:** 2009-02-11

**Authors:** Hong-Tao Xu, Jorge L Martinez-Cajas, Michel L Ntemgwa, Dimitrios Coutsinos, Fernando A Frankel, Bluma G Brenner, Mark A Wainberg

**Affiliations:** 1 McGill University AIDS Centre, Lady Davis Institute, Jewish General Hospital, Montreal, Quebec H3T1E2, Canada; 2 Department of Medicine, McGill University, Montreal, Quebec H3A 2T5, Canada; 3 Department of Microbiology and Immunology, McGill University, Montreal, Quebec H3A 2T5, Canada

## Abstract

**Background:**

We investigated the effects of mutations K65R and K65R plus M184V on enzymatic function and mechanisms of drug resistance in subtype C reverse transcriptase (RT).

**Methods:**

Recombinant subtype C HIV-1 RTs containing K65R or K65R+M184V were purified from *Escherichia coli*. Enzyme activities and tenofovir (TFV) incorporation efficiency by wild-type (WT) and mutant RTs of both subtypes were determined in cell-free assays. Efficiency of (-) ssDNA synthesis and initiation by subtype C RTs was measured using gel-based assays with HIV-1 PBS RNA template and tRNA3^Lys ^as primer. Single-cycle processivity was assayed under variable dNTP concentrations. Steady-state analysis was performed to measure the relative inhibitory capacity (ki/km) of TFV-disphosphate (TFV-DP). ATP-dependent excision and rescue of TFV-or ZDV-terminated DNA synthesis was monitored in time-course experiments.

**Results:**

The efficiency of tRNA-primed (-)ssDNA synthesis by subtype C RTs was: WT > K65R > K65R+M184V RT. At low dNTP concentration, K65R RT exhibited lower activity in single-cycle processivity assays while the K65R+M184V mutant showed diminished processivity independent of dNTP concentration. ATP-mediated excision of TFV-or ZDV-terminated primer was decreased for K65R and for K65R+M184V RT compared to WT RT. K65R and K65R+M184V displayed 9.8-and 5-fold increases in IC50 for TFV-DP compared to WT RT. The Ki/Km of TFV was increased by 4.1-and 7.2-fold, respectively, for K65R and K65R+M184V compared to WT RT.

**Conclusion:**

The diminished initiation efficiency of K65R-containing RTs at low dNTP concentrations have been confirmed for subtype C as well as subtype B. Despite decreased excision, this decreased binding/incorporation results in diminished susceptibility of K65R and K65R+M184 RT to TFV-DP.

## Background

The human immunodeficiency virus type 1 (HIV-1) epidemic has rapidly evolved to include 6 major circulating subtypes (A, B, C, D, G, F) and numerous recombinant forms, showing 25–35% overall genetic variation, including 10–15% in reverse transcriptase (RT) [1-3]. The RT enzyme naturally exists as a p66/p51 heterodimer that can undergo post-translational modification in terms of its presence in both virions and cells [4]. Subtype C variants of HIV-1 are responsible for ~50% of the worldwide pandemic, representing the dominant epidemics in Sub-Saharan Africa and India [5]. In spite of this, no work has yet been reported on the differential biochemistry of subtype C reverse transcriptase (RT). Most data are inferred from enzymatic studies on prototypic subtype B viruses circulating in the Western world that represent < 12% of the global pandemic [5].

Genetic divergence in the RT enzyme may also be linked to differential acquisition of resistance to nucleoside or nucleotide RT inhibitors (N(t)RTIs) that are core constituents of antiretroviral (ARV) regimens for treatment of HIV-1 infection. These drugs include the eight N(t)RTIs approved for clinical treatment of HIV-1 infection: zidovudine (ZDV), stavudine (d4T), didanosine (ddI), lamivudine (3TC), zalcitabine (ddC), abacavir (ABC), emtricitabine (FTC) and tenofovir disoproxil fumarate (TDF) [6].

The RT mutation K65R can be selected by each of tenofovir (TFV), ddI, ddC, ABC and d4T and yields decreased susceptibility to all clinically used NRTIs except ZDV [7-9]. Our laboratory has described the facilitated selection of K65R in subtype C in cell culture [10]. Recent clinical studies show the preferential emergence of K65R in subtype C-infected patients failing d4T/ddI based regimens in Botswana (30%), and d4T/3TC-based regimens in South Africa and Malawi (7–20%) [11-13]. In contrast, K65R is present in only 1.8% of subtype B HIV-1 infected patients failing d4T based regimens in the Stanford HIV Resistance Database (accessed Dec 11, 2008) and is only common in patients failing TFV-containing regimens (up to15%) [14-17].

Although subtype C viruses harbour a unique KKK nucleotide motif, amino acid polymorphisms and codon bias at position 65 cannot explain the differential acquisition of K65R in subtype C variants. In subtype B the mutation required in codon 65 is AAA → AGA while it is AAG → AGG in subtype C. The present study was designed to determine if variations in enzymatic function might be responsible for the higher propensity of K65R to occur in subtype C. In this work, we have characterized the enzymatic properties of recombinant B and wild-type RTs as well as RTs harboring the K65R and K65R/M184V mutations.

## Results

### Purification of recombinant HIV-1 RT and specific activity analysis

**Figure 1:**
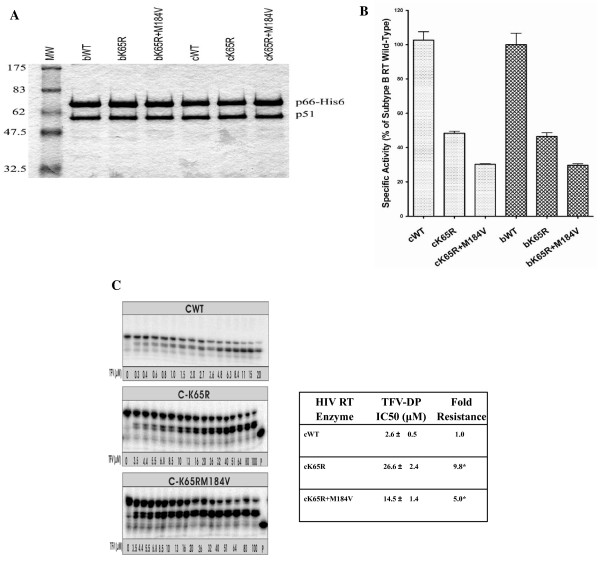
**Purification, determination of specific activity and TFV susceptibility of recombinant subtype C and B HIV-1 RTs**. (A) Coomassie-Brilliant Blue staining of purified heterodimer RTs after 8% SDS-PAGE. MW (molecular mass standards in kilo daltons are shown on the left); b/cWT, (subtype B/C HIV-1 RT wild-type); b/cK65R, (subtype B/C HIV-1 RT harboring K65R); b/cK65R+M184V, (subtype B/C HIV-1 RT harboring K65R+M184V). The positions of purified recombinant RT heterodimers are indicated on the right. (B) Specific activity of recombinant RT enzymes as assessed using poly(rA)/oligo(dT) template/primer as described in Materials and Methods. All specific activities are expressed as a percentage of subtype B wild-type RT specific activity. (C) Incorporation efficiency of TFV-DP by subtype C WT and mutant RTs was monitored by gel-based assay and a representative image is shown in the panel on the left. Primer 19D was 5'-end labeled and annealed to template 57D. Reactions were performed with increasing concentrations of TFV-DP. P indicates the position of 5'-end labeled primer. Fifty percent inhibitory concentration (IC^50^) and fold resistance are shown on the right. Values are means of at least three independent experiments ± standard deviation. *P ≤ 0.05 compared to the IC^50 ^of wild-type, by two-tailed Student's *t*-test.

Recombinant heterodimer (p66/p51) RTs from both subtype C and B were purified to > 95% homogeneity; all RT subunits p66 and p51 were processed to similar molar ratio (Fig. 1A). To determine the specific activity of the recombinant enzyme preparations, DNA polymerase activity was measured using synthetic poly(rA)/(dT)12–18 template/primer over a 15-min initial rate reaction. The calculated initial velocities were then divided by the concentration of enzyme used in the assay to determine the specific activity of the recombinant RT preparations (Fig. 1B). Wild-type RTs from both subtypes shared similar activities. All mutant enzymes were significantly impaired in specific activity compared with wild-type enzyme, with K65R exhibiting only 46%–50% of wild-type activity and K65R+M184V RT exhibiting only ≈ 30% of wild-type activity. The observation of diminished activity associated with K65R mutant RTs of both subtypes is in agreement with results obtained previously with subtype B K65R RT [18].

### Tenofovir susceptibility in cell-free assays

Previous cell culture assays showed that viruses of subtypes A/E, B, C harboring K65R exhibited similar 6.5 to 10-fold resistance to TFV [10]. In this study, we determined the efficiencies of incorporation of TFV-DP using subtype C WT and mutant K65R and K65R+M184V RTs in gel-based assays using the 19D/57D primer/template system (FIG. 1C, left). Calculations of IC50s for TFV-DP showed that subtype C K65R RT displayed a 9.8-fold decreased susceptibility to TFV-DP compared with WT RT. The simultaneous presence of K65R and M184V resensitized these enzymes for TFV-DP by 5-fold compared to WT RT (FIG. 1C, right). As a result, the order of susceptibility of subtype C RTs to TFV-DP was WT > K65R+M184V > K65R. These results are in good agreements with those obtained with subtype B HIV-1 recombinant RTs [19].

### Efficiency of (-)ssDNA synthesis

**Figure 2:**
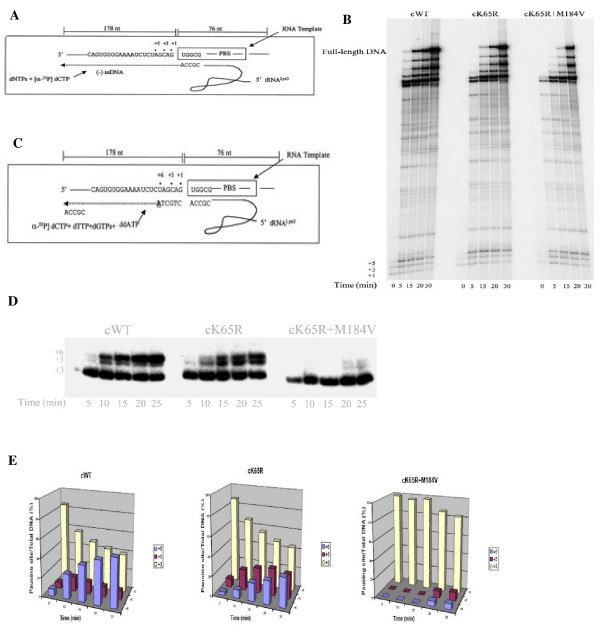
**Efficiency of (-)ssDNA synthesis in cell-free assay**. The efficiencies of the reactions with WT and mutant RTs were compared in time course experiments. (A) Graphic representation of the cell-free system (HIV-1 PBS RNA/tRNA^3Lys^) used to monitor the synthesis of (-)ssDNA. (B) Synthesis of full-length DNA by WT and mutant enzymes. Reactions were initiated with 10 μM dNTPs and monitored by incorporation of [α-^32^P]-dCTP. Full-length DNA product and pausing sites are shown on the left. (C) Graphic representation of the cell-free system (HIV-1 PBS RNA/tRNA^3Lys^) used to monitor the efficiency of initiation of (-)ssDNA synthesis in the presence of the chain-terminator ddATP. (D) Initiation of (-) ssDNA synthesis by WT and mutant enzymes. Reactions were performed using 1 μM dNTPs, and ddATP was employed in place of dATP to give rise to a six-nucleotide initiation product. ddATP-terminated +6 product and +3 and +5 pausing position are shown on the left side. (E) Graphic representation of the gel-based assays shown in D.

The reduced efficiency of initiation of (-)ssDNA synthesis and tRNA primer usage, associated with subtype B RTs harboring K65R and K65R+M184V is a mechanism responsible for the diminished replicative fitness of viruses containing these substitutions (Fig 2A) [18]. In cell culture assays, subtype C K65R viruses, like subtype B K65R viruses, exhibited lower replication capacity and addition of M184V enhanced this effect [10]. In our cell-free assay with subtype C RTs harbouring K65R and K65R/M184V, we also observed impaired efficiency of (-)ssDNA synthesis; the decrease in product formation was most pronounced at earlier time points (Fig. 2B). Mutant K65R/M184V RT displayed maximal decrease in product formation and accumulation at the +3 and +5 pausing sites. The order of efficiency of (-)ssDNA synthesis was WT > K65R >> K65R+M184V. In pilot time-course experiments, we also performed reactions at high dNTP concentration (100 μM-200 μM), and observed that the double mutant enzyme showed reduced efficiency in ssDNA synthesis; in contrast K65R RT showed similar efficiency as WT (data not shown). To further analyze changes in pausing patterns, we modified the assay described above and restricted DNA synthesis to the initiation stage by limiting dNTPs to 1 μM and addition of ddATP at position +6 (Fig. 2C). The results in Fig. 2D and Fig. 2E show that release from the pausing site at position +5 was compromised with K65R RT, while the K65R+M184V RT was severely impaired in release from the +3 pausing site. These observations are similar to those reported with subtype B RTs [19].

### Single-cycle processivity of subtype C RTs

**Figure 3:**
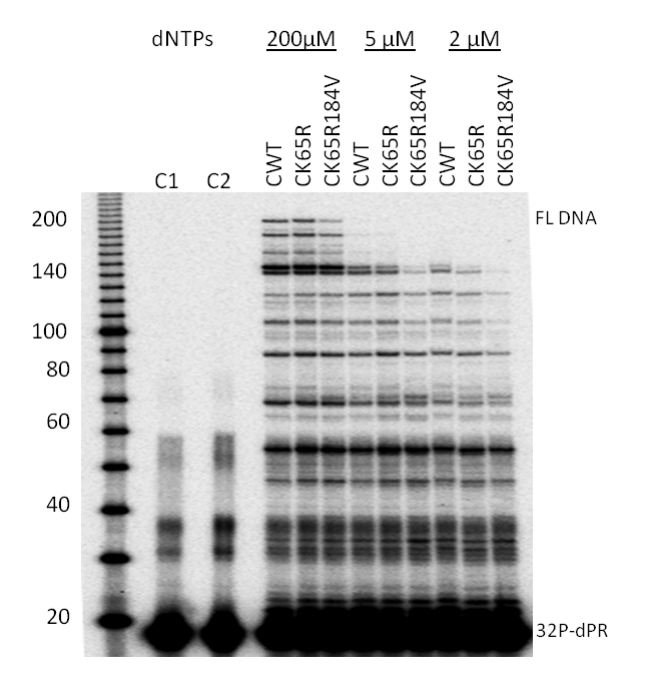
**dNTP concentration dependence of single-cycle processivity of WT and mutant RTs**. The DNA primer dPR was 5'-end labeled with [γ-^32^P]ATP and annealed to HIV PBS RNA. Extension was performed using a heparin trap and equivalent amounts of recombinant RTs at three different dNTP concentrations: 200 μM, 5 μM, and 2 μM. The sizes of some fragments of the ^32^P-labeled 10 bp DNA ladder (Invitrogen) in nucleotide bases are shown on the left. Positions of ^32^P-labeled dPR primer (^32^P-dPR) and full-length extension product (FL DNA) are indicated on the right.

Analyses of single-cycle processivity were performed with HIV PBS RNA and 5'-end labeled dPR primer under variable dNTP concentrations with heparin as a trap. The products of this primer extension assay were separated on a 6% PAGE-7M urea sequencing gels and subjected to phosphorimager analysis (FIG. 3). At high dNTP concentration (200 μM), K65R RT showed similar activity as WT, while the double mutant K65R+M184V RT was impaired in primer extension. As dNTP concentration decreased, K65R RT showed less extension than WT enzyme; the difference was more pronounced in reactions with the lowest dNTP concentration. Similar results were obtained with subtype B RT WT and K65R and K65R+M184V mutant RTs (data not shown).

### Relative binding/incorporation of dATP and TFV-DP by subtype C RT enzymes

One mechanism of resistance to NRTI is decreased binding or incorporation of inhibitor relative to natural substrate. To determine the effects of mutations K65R and K65R+M184V in subtype C RTs on TFV-binding and incorporation, we measured the steady-state kinetic constant Km for dATP and inhibition constant Ki for TFV-DP (Table 1). The steady state Km value of K65R RT for dATP was slightly elevated (0.51 μM to 0.64 μM) compared to WT RT, suggesting that subtype C K65R RT binds to and incorporates the natural dATP substrate with an efficiency similar to or slightly reduced to that of WT RT. However, the Ki value of K65R RT for TFV-DP was significantly increased compared to that of WT (*P *≤ 0.01). Thus, the relative inhibitory capacity (Ki/Km) for TFV-DP was increased by 7.2-fold compared to WT. For the double mutant K65R/M184V, the Km value for dATP was significantly increased compared to that of WT (*P *≤ 0.01) and the Ki value for TFV-DP was also increased compared to WT (*P *≤ 0.01). Ki/Km was elevated by 4.1-fold compared to WT. These results are in agreement with published data obtained with subtype B RTs [9].

**Table 1:** Steady state kinetic analysis for dATP and TFV-DP: measurement of relative inhibitory capacity (Ki/Km ratio)

HIV-1 RT Enzyme	Km(dATP), μM^a^	Ki(TFV), μM^b^	Ki/Km(fold)^c^
Subtype C WT	0.51 ± 0.04	0.24 ± 0.04	0.47(1.0)
Subtype C K65R	0.64 ± 0.05	2.2 ± 0.07*	3.43(7.2)
Subtype C K65RM184V	0.83 ± 0.04*	1.2 ± 0.05*	1.93(4.1)

^a ^Km and ^b ^Ki values are mean values from at least three experiments ± SD.

^c ^Fold change in Ki/Km from wild-type.

* *P *≤ 0.01 compared to the wild-type by two-tailed Student's *t*-test.

### Efficiency of ATP-dependent excision of NRTIs and rescue of DNA synthesis

**Figure 4:**
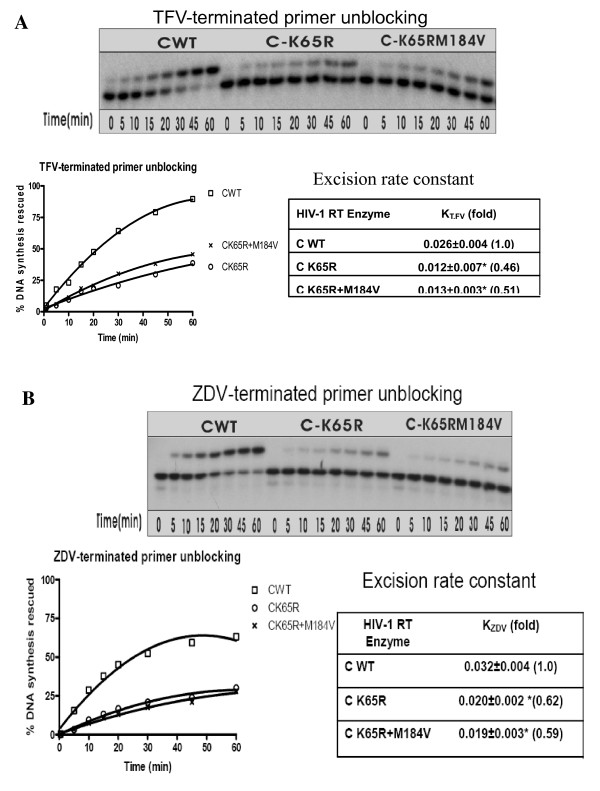
**ATP-dependent excision of chain-terminating nucleotides with WT and mutant RTs**. The primers were initially chain terminated with TFV-DP (A) or ZDV-MP (B). Combined excision/rescue reactions were compared in time course experiments. Reactions were stopped at the indicated time points and samples were analyzed in denaturing 6% polyacrylamide gels. Graphic representations of efficiency of rescued DNA synthesis from gel-based assays are shown on the left below the gel graph. Calculated excision rate constants (k) (×10^-3 ^s^-1^) ± SD (*P *≤ 0.01, compared to WT; two-tailed Student's *t*-test) are shown on the right.

Excision of incorporated NRTIs is a second mechanism of NRTI resistance by mutant RTs. Using the subtype C RT enzymes, we determined the excision efficiency of TFV and ZDV-MP using gel-based ATP-dependent excision experiments in the presence of fixed concentrations (10 μM) of the next complementary nucleotide as described [19,20]. For both the TFV-(Fig. 4A) and ZDV-(Fig. 4B) terminated primers, the subtype C WT RT mutants K65R and K65R+M184V RT showed impaired excision efficiency compared with WT. ATP-mediated excision of TFV-or ZDV-terminated primer was decreased by 2.6-and 3.1-fold for K65R and K65R+M184V RTs, respectively (TFV 23%, ZDV 15% at 30 min) compared to WT RT (TFV 60%, ZDV 47% at 30 min). Initial excision rate constants showed that TFV and ZDV-MP were more stable when incubated with the mutant enzymes.

## Discussion

Our experiments have revealed that subtype C HIV-1 RT has similar enzymatic activity to subtype B RT, and that the K65R and K65R+M184V mutations, affect subtype C RT function in a manner similar to that seen with subtype B RT. Specific effects include: 1) The efficiency of ssDNA synthesis and initiation is reduced; 2) At low dNTP concentration, K65R RT exhibited lower activity in single-cycle processivity assays while the K65R+M184V mutant showed diminished processivity independent of dNTP concentration. 3) the discrimination of nucleotides is equivalently reduced in subtype C RT as in subtype B RT; and 4) the excision of incorporated nucleotides is also decreased in a similar fashion in both RTs, in agreement with previous results [9,19,21-23]. We also confirm that the biochemical basis for the HIV-1 fitness loss that results from the acquisition of the K65R and K65R/M184V mutations are also valid in HIV-1 subtype C RT. The same TFV resistance mechanisms exist in both subtypes B and C, and both impaired discrimination and excision determine TFV susceptibility.

The K65R mutation is located in the fingers domain of RT and its effect on reduction of NRTI incorporation and reduced excision is probably due to an increased rigidity of the active site and effective trapping of the dinucleoside tetraphosphate excision product [24]. Natural polymorphisms within subtype C RT did not alter either the direction or the magnitude of the effect of the resistance mutations K65R and M184V. The subtype C RT used in our experiments contains 33 amino acid polymorphisms that are different from the subtype B consensus sequence. Only the polymorphisms at positions 35, 36, 48 (in the fingers), 211, 214 (in the palm), and 245, 286 and 291 (in the thumb) are located close enough to the RT active site to have significant functional interactions with the fingers. However, such effects do not appear to be discernible by the methods used in our study. Hence, the overall effect of K65R in subtype C is to reduce susceptibility to TFV.

In the absence of biochemical evidence of an enzyme-dependent mechanism for the preferential emergence of K65R in HIV-1 subtype C, the possibility of a template-dependent mechanism is favoured as described by our laboratory elsewhere [25]. Briefly, it seems that increased pausing is involved when RT copies a HIV-1 subtype C nucleic acid template at RT positions 64 through 66, due to the combined effect of low fidelity and NRTI pressure. This was shown to be true for reactions involving RT of either subtype B or C origin but only with template C sequences [25]. Further virological tests, including competition assays, are warranted in order to detect more subtle effects of the K65R mutation in subtype C.

Based on standard genetic sequencing of HIV-1 RNA from plasma of treated patients, K65R and M184V can emerge in subtype C as in B viruses after therapeutic failure with ABC, ddI, TFV and d4T when combined with 3TC. The finding that both K65R and K65R/M184V decrease the enzymatic fitness of RT in subtype C HIV and that both restore susceptibility to ZDV in an additive manner is important in view of the ongoing switch in use of NRTI backbones away from thymidine analogs and the higher frequency of K65R in subtype C isolates from African patients [11-13]. Newer backbones (TFV/FTC and ABC/3TC) typically select for the K65R and M184V mutations in HIV-1 subtype B, but these mutations have also frequently emerged in subtype C viruses treated with d4T/3TC. As described here, these mutations cause loss of enzymatic fitness and might reduce the virulence of HIV-1.

Studies with both SIV and a RT SHIV in macaques treated with TFV monotherapy showed selection of the K70E and K65R mutations [26,27]. The viral load of the animals with virologic failure were about 10-fold below the pre-therapy set point, which might be related to loss of viral fitness [26]. Interestingly, the presence of TFV resistance mutations did not preclude virological suppression in several of the treated animals [26]. A CD8-mediated immunologic response seemed to contribute to virologic suppression in animals harboring TFV-resistant viruses, but this only occurred if TFV was continuously administered.

We did not test whether thymidine analog resistance mutations (TAMs) affect subtype C RT enzymatic function. Therefore, we cannot comment on the extent to which TAMs might affect TFV incorporation or excision. However, research on this matter is warranted because TAMs also occur in NRTI-resistant subtype C viruses isolated from patients who have failed first line regimens in resource-limited settings [28-30].

## Conclusion

Our results show that an enzyme-based mechanism is not the basis for the higher propensity of HIV-1 subtype C to acquire the K65R mutation in response to NRTI exposure and that subtype B and C RTs behave similarly in regard to most enzymatic properties. In particular, both enzymes, when containing K65R, share a diminished initiation efficiency at low dNTP concentrations as well as diminished rates of excision if K65R is present. Both subtype B and subtype C RTs containing K65R are less able to bind to TFV-DP and are less susceptible than WT RTs to the chain-terminating effects of this compound.

## Methods

### Chemicals and Nucleic Acids

Tenofovir diphosphate (TFV-DP) was kindly provided by Gilead Sciences (Foster City, California, USA). Zidovudine triphosphate (ZDV-TP) was purchased from Trilink Biotechnologies (San Diego, California, USA). Poly(rA)/oligo(dT)12–18 ultrapure dNTPs, NTPs and ddATP were purchased from GE Healthcare. [^3^H] dTTP (70–80 Ci/mmol) was from Perkin Elmer Life Sciences. [α-^32^P]dNTPs and [γ-^32^P]ATP were obtained from MP Biomedicals.

Natural human tRNA3^Lys^purified from placenta by high-pressure liquid chromatography (HPLC) was purchased from BIO S&T (Montreal, Quebec, Canada). The DNA primer/template (P/T) substrates used for measuring efficiency of chain-termination of TFV-DP and ATP-mediated primer unblocking were derived from the polypurine tract (PPT) of the HIV-1 genome [30] and were: 57D(5'-GTTGGGAGTGAATTAGCCCTTCCAGTCCCCCCTTTTCTTTTAAAAAGTGGCTAAGA-3' 17D 5'-TTAAAAGAAAAGGGGGG-3' 19D 5'-TTAAAAGAAAAGGGGGGAC-3'

An HIV-1 RNA template spanning the 5' UTR to the primer binding site (PBS), was *in vitro *transcribed from BSSH II-linearized pHIV-PBS DNA by using T7-Megashortscript kit (Ambion, Austin, TX) as described [31]. For preparation of subtype C HIV-1 PBS RNA template, plasmid pHIV-c-PBS was first constructed by Pst I-Bgl II digestion of the 1.4 kb PCR amplification product with primers CLTRF 5'-GGAAGGGTTAATTTACTCTAAGAAAAGGC-3' and CLTRPstIR 5'CTATCCCATTCTGCAGCCTCCTCA-3' and MJ4 DNA template; the resulting 0.9 kb fragment was subcloned into the pSP72 vector DNA fragment linearized with the same enzymes; transcription was performed as above after Pvu II linearization.

### Recombinant Reverse Transcriptase Expression and Purification

The subtype B HIV-1 RT expression plasmid pRT6H-PROT [24] was kindly provided by Dr. S. F. J. Le Grice. Subtype B RTs containing mutations K65R and K65R+M184V were generated as described previously [19]. For construction of subtype C HIV-1 RT from the heterodimer expression plasmid pcRT6H-PROT, the RT coding region of subtype C HIV-1 isolate BG05 (GenBank accession number AF492609) was subcloned into pRT6H-PROT by standard PCR cloning procedure to replace the subtype B RT coding region [32]. Mutant DNA constructs K65R and K65R+M184V were generated by Quick-change Mutagenesis Kit (Strategene). The presence of mutations and accuracy of the RT coding sequence was verified by DNA sequencing. Polymorphisms within subtype C RT differ from subtype B as follows: V35T, T39E, S48T, K166R, K173T, D177E, T200A, Q207E, R211K, L214F, V245K, T286A, E291D, I293V, R356K, G359T, T376A, T377Q, K390R, T403A, E404D, V435P, A437V, N460D, V466I, T468S, D471E, Y483Q, L491S, Q512K, K527Q, K530R, A534S. Recombinant wild-type (WT) and mutated RTs were expressed and purified as described [33,34]. In brief, RT expression in bacteria *Escherichia coli *M15 (pREP4) (Qiagen) was induced with 1 mM isopropyl-*b*-D-thiogalactopyranoside (IPTG) at room temperature. The pelleted bacteria were lysed under native conditions with BugBuster Protein Extraction Reagent (Novagen), clarified by high speed centrifugation, and the supernatant was subjected to the batch method of Ni-NTA metal-affinity chromatography using QIA *expressionist *(Qiagen) according to the manufacturer's specifications. All buffers contained complete protease inhibitor cocktail (Roche). Histidine-tagged RT was eluted with an imidazole gradient. RT-containing fractions were pooled, passed through DEAE-Sepharose (GE Healthcare), and further purified using SP-Sepharose (GE Healthcare). Fractions containing purified RT were pooled, dialyzed against storage buffer (50 mM Tris [pH 7.8], 25 mM NaCl and 50% glycerol), and concentrated to 2 mg 4 mg/ml with Centricon Plus-20 MWCO 30 kDa (Millipore). Protein concentration was measured by Bradford Protein Assay kit (Bio-Rad Laboratories) and the purity of the recombinant RT preparations was verified by SDS-PAGE.

### Specific activity determination

The RNA-dependent DNA polymerase activity of each recombinant RT preparation was assayed in duplicate using poly(rA)/p(dT)12–18 template/primer (GE Healthcare) as described [17,33]. Each 50-μl reaction contained 25 μg/ml poly(rA)/p(dT)12–18, 50 mM Tris (pH 7.8), 5 mM MgCl2, 60 mM KC1, 10 mM dithiothreitol (DTT), 5 *μ*M dTTP with 2.5 *μ*Ci of [^3^H]dTTP and variable amounts of wild-type or mutated RT. Reactions were performed at 37 w and aliquots of 15 ul were removed at 3 min, 9 min, 15 min and quenched with 0.2 ml of 10% cold trichloractic acid (TCA) and 20 mM sodium pyrophosphate. After 30 min on ice, the precipitated products were filtered onto 96-well plates using glass fiber filters (Millipore) and sequentially washed with 10% TCA and 95% ethanol. The radioactivity of incorporated products was analyzed by liquid scintillation spectrometry. The incorporated [^3^H] dTTP was plotted as cpm versus time and initial velocities were determined from the slopes of the linear regression analyses using GraphPad Prism 4.0 software. Specific activities were calculated as described previously [18]. All values are presented as a percentage of specific activity of subtype B WT RT with the percentage standard deviation of the duplicate samples also indicated.

### Incorporation efficiency of TFV-DP in cell-free assay

Incorporation of TFV-DP was monitored using 19D/57D primer/template system as described for measurement of ddATP incorporation [31,35]. Inhibition efficiency was expressed as the concentration of TFV producing a 50% inhibition (IC50) of full-length DNA synthesis.

### Determination of steady-state kinetic parameters

The Km for dATP and the K*i *for TFV-DP were determined by filter binding assays as described previously [36]. In brief, 200 nM dPR were heat-annealed to 300 nM subtype C HIV-1 PBS RNA in a buffer containing 50 mM Tris-HCl pH 7.8 and 50 mM NaCl. The pre-hybridized primer-template complex was mixed with variable amounts of WT or mutated RT in the presence of 5 mM MgCl2, 5 mM dithiothreitol, 50 μM dCTP/dGTP/dTTP, 200–500 nCi of [^3^H]dATP (> 70–80 Ci/mmol), 5 U of RNase inhibitor and variable concentrations of dATP in the absence or presence of TFV-DP. Reactions were incubated at 37°C. Aliquots were removed at 3 min, 7 min, 15 min and quenched with 10% trichloroacetic acid (TCA) and 20 mM sodium pyrophosphate. After 30 minutes on ice, the precipitated products were filtered onto 96-well glass fibre filter plates (Millipore), washed twice with 10% TCA and once with 95% ethanol. Incorporated radioactivity was measured by liquid scintillation counting. Kinetic constants were determined using Graphpad Prism 4.0 software as described [36].

### Efficiency of synthesis of minus-strand strong stop DNA [(-)ssDNA]

The efficiency of (-)ssDNA synthesis was determined by cell-free assay as described [19,31,37]. Briefly, 20 nmol/l tRNALys3 were heat annealed to 40 nmol/l PBS RNA. Then, 100 nmol/l WT or mutated RTs and 6 mmol/l MgCl2 were added. Reactions were initiated with 10 μM dNTPs and monitored by incorporation of [α-^32^P]-dCTP.

Aliquots were removed at various time points and quenched with 95% formamide-40 mM EDTA. Samples were resolved in 6% polyacrylamide-7M urea gel and analyzed by using the Molecular Dynamics Typhoon Phosphorimager system (GE Healthcare). To study the effect of mutated RTs on the initiation of synthesis of (-)ssDNA, the above reactions were initiated with 1 μM dNTPs, except ddATP was employed as a termination nucleotide instead of dATP to give rise to a six-nucleotide initiation product. Products were separated as described above and analyzed by ImageQuant software.

Using the same gel-based system as described [19,37], we evaluated the efficiency of initiation of (-)ssDNA synthesis by subtype C WT RT and mutant RTs harboring mutations K65R and K65R/M184V. The preannealed human tRNA3^Lys ^– HIV PBS RNA complexes were incubated with either WT or mutant RT enzymes to initiate the RT reaction in the presence of 10 μM dNTPs. Time-course experiments were performed, and products were separated and analyzed by ImageQuant software as described above.

### Single-cycle processivity assays

The 18-nt DNA primer dPR complementary to the viral PBS was 5'end labeled using [γ-^2^P]ATP. The dPR primer (500 nM) containing labeled dPR as tracer was annealed to PBS RNA transcript. RT (50 nM) was then preincubated with the T/P for 5 min at 37°C before initiation of the reaction by the addition of dNTPs using a heparin trap (1 mg/ml). Three concentrations of dNTPs were assayed: 200 μM, 5 μM and 2 μM. After 30 min of incubation at 37°C, aliquots of the reaction mixtures were removed and quenched with 95% formamide-40 mM EDTA. The samples were heated at 100°C for 5 min, then analyzed by 6% polyacrylamide-7M urea gel. Resolved products were analyzed by phosphorimager.

### Excision and rescue of chain-terminated DNA synthesis in the presence of ATP

To generate TFV- or ZDV-terminated primers, primers 17D and 19D were first radiolabeled at the 5' end and subsequently extended with TFV-DP and ZDV-TP respectively using cWT RT and annealed to template oligonucleotide 57D as described [38]. Excision and the ensuing rescue of chain-terminated DNA synthesis were monitored as described [19,21]. Time course experiments were performed after the addition of 3.5 mM ATP (pretreated with inorganic pyrophosphatase) and a dNTP cocktail consisting of 100 μM dATP, 10 μM dCTP, and 100 μM ddTTP for TFV and 100 μM dTTP, 10 μM dCTP, and 100 μM ddGTP for ZDV. Samples were resolved in an 6% polyacrylamide7M urea gel followed by phosphorimaging. Band intensities were analyzed by ImageQuant softaware. Initial excision rate constants (k) were determined as described previously using SigmaPlot 9.0 [39].

## Competing interests

The authors declare that they have no competing interests.

## Authors' contributions

HX performed experiments and drafted the manuscript. JLM-C aided in drafting the manuscript. MLN performed sequencing reactions. DC performed experiments and aided in drafting the manuscript. FAF performed sequencing experiments. BGB aided in drafting the manuscript. MAW supervised the project, aided in drafting the manuscript, and provided resources for the research.
